# Development of a Bifunctional Andrographolide-Based Chemical Probe for Pharmacological Study

**DOI:** 10.1371/journal.pone.0152770

**Published:** 2016-04-01

**Authors:** Ya-Hsin Hsu, Yu-Ling Hsu, Sheng-Hung Liu, Hsin-Chia Liao, Po-Xuan Lee, Chao-Hsiung Lin, Lee-Chiang Lo, Shu-Ling Fu

**Affiliations:** 1 Institute of Traditional Medicine, National Yang-Ming University, Taipei, Taiwan; 2 Department of Chemistry, National Taiwan University, Taipei, Taiwan; 3 Department and Institute of Pharmacology, National Yang-Ming University, Taipei, Taiwan; 4 Program in Molecular Medicine, National Yang-Ming University and Academia Sinica, Taipei, Taiwan; 5 Department of Life Sciences and Institute of Genome Sciences, National Yang-Ming University, Taipei, Taiwan; Stanford University, UNITED STATES

## Abstract

Andrographolide (ANDRO) is a lactone diterpenoid compound present in the medicinal plant *Andrographis paniculata* which is clinically applied for multiple human diseases in Asia and Europe. The pharmacological activities of andrographolide have been widely demonstrated, including anti-inflammation, anti-cancer and hepatoprotection. However, the pharmacological mechanism of andrographolide remains unclear. Therefore, further characterization on the kinetics and molecular targets of andrographolide is essential. In this study, we described the synthesis and characterization of a novel fluorescent andrographolide derivative (ANDRO-NBD). ANDRO-NBD exhibited a comparable anti-cancer spectrum to andrographolide: ANDRO-NBD was cytotoxic to various types of cancer cells and suppressed the migration activity of melanoma cells; ANDRO-NBD treatment induced the cleavage of heat shock protein 90 (Hsp90) and the downregulation of its client oncoproteins, v-Src and Bcr-abl. Notably, ANDRO-NBD showed superior inhibitory effects to andrographolide in all anticancer assays we have performed. In addition, ANDRO-NBD was further used as a fluorescent probe to investigate the uptake kinetics, cellular distribution and molecular targets of andrographolide. Our data revealed that ANDRO-NBD entered cells rapidly and its fluorescent signal could be detected in nucleus, cytoplasm, mitochondria, and lysosome. Moreover, we demonstrated that ANDRO-NBD was covalently bound to several putative target proteins of andrographolide, including NF-κB and hnRNPK. In summary, we developed a fluorescent andrographolide probe with comparable bioactivity to andrographolide, which serves as a powerful tool to explore the pharmacological mechanism of andrographolide.

## Introduction

**Fig 1:**
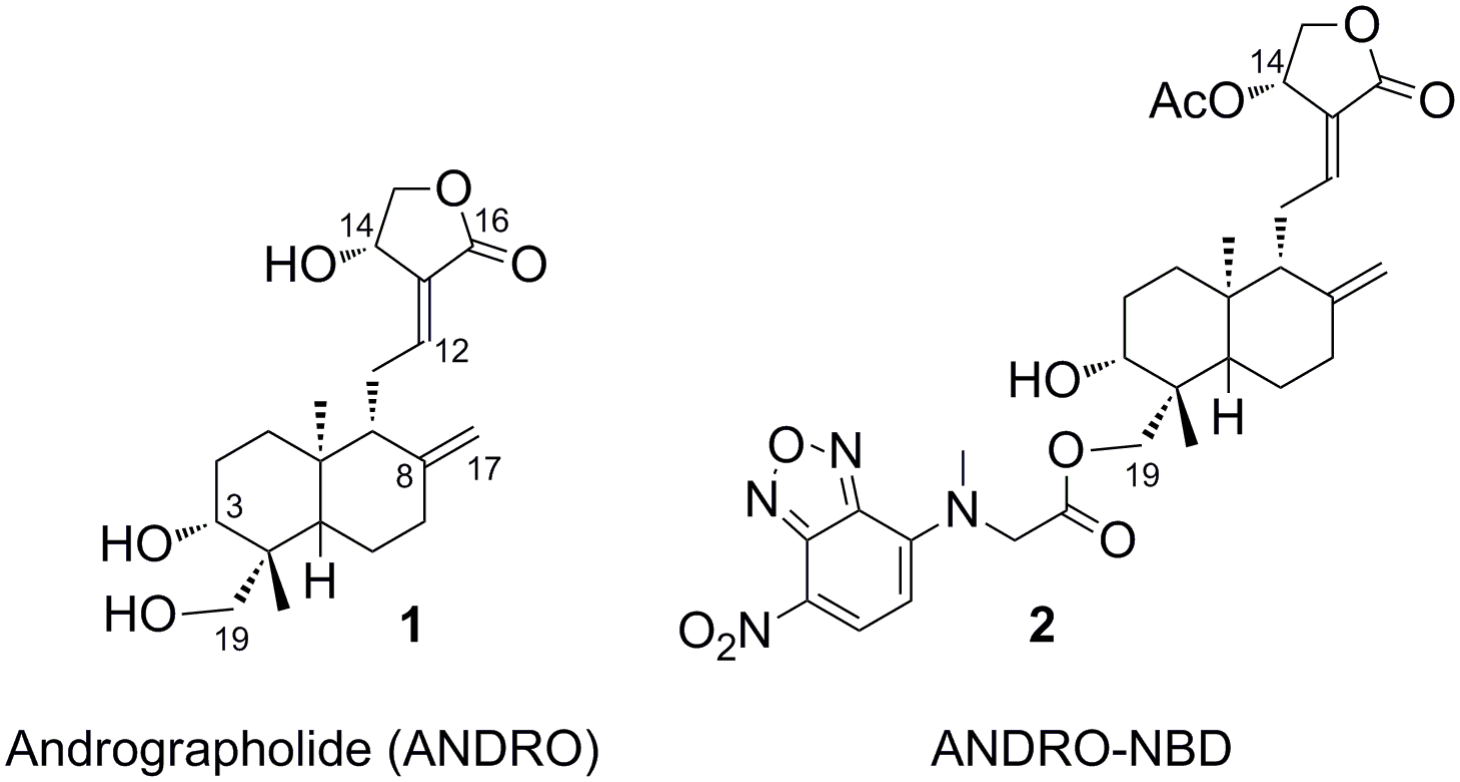
Structures of andrographolide (ANDRO, 1) and an NBD-conjugated andrographolide derivative 2 (ANDRO-NBD).

Andrographolide (structure **1** in Fig 1) is the major active ingredient extracted from the leaves of *Andrographis paniculata* which has been used as a medicinal herb in Asia and Europe [1, 2]. Andrographolide and its derivatives have been shown to exhibit a broad spectrum of pharmacological activities, including anti-inflammatory [2], antiviral [3], anticancer [4], antidiabetic [5], and hepatoprotective effects [6]. However, the detailed molecular mechanism underlying these activities awaits further investigation, which prompts us to develop an andrographolide-based chemical probe.

Andrographolide featuring an exocyclic ene-γ-lactone moiety, belongs to member of electrophilic natural products that can form a covalent bond with their targets through Michael addition reaction [7]. In fact, andrographolide has been demonstrated to inhibit the transcriptional activity of NF-κB through forming a covalent adduct with p50 subunit [8, 9]. Currently, the ability to form covalent bonds has made electrophilic natural products an attractive molecular framework for probe development in modern chemical biology [10]. In a typical design of chemical probes, a suitable fluorophore is attached to the natural product at a position that would not drastically alter the bioactivity of the parent compound. To date, several andrographolide-based probes have been synthesized [8, 9, 11], but no bioactive fluorophore-conjugated andrographolide derivative has been developed. We herein report the synthesis, biological activities, and labeling performance of a fluorescent andrographolide-derived chemical probe (structure **2** in Fig 1; designated as ANDRO-NBD in subsequent context). Development of such a fluorescent derivative that retains the characteristic bioactivities of andrographolide would be of great value in studying pharmacological mechanism of andrographolide.

## Materials and Methods

### Chemicals and Synthesis of ANDRO-NBD

**Fig 2:**
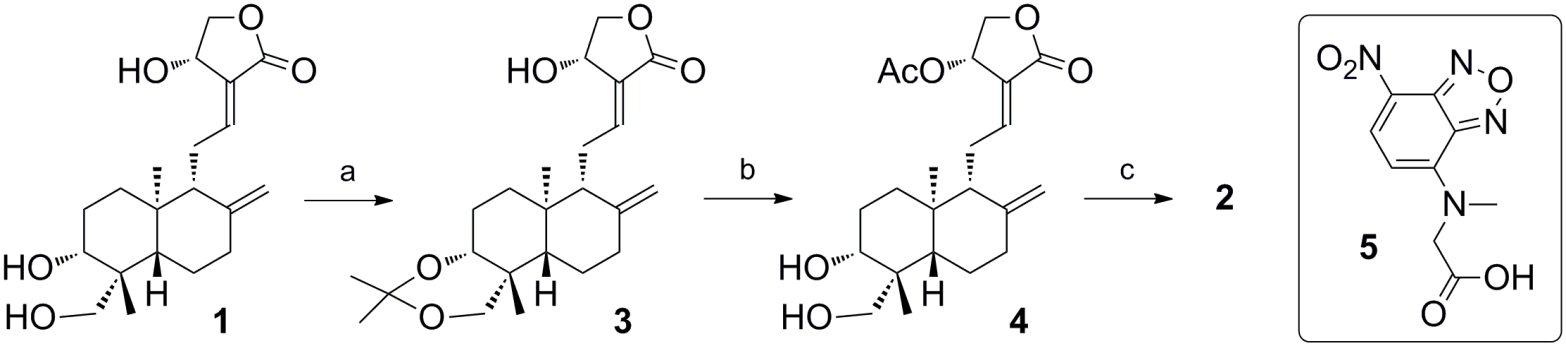
Synthesis of 2 (ANDRO-NBD). *Reagents and conditions*: (a) 2,2-dimethoxypropane, PPTS, acetone, 30 min, 97%; (b) (i) Ac_2_O, reflux for 1.5 h, (ii) AcOH/H_2_O (7:3), 10 min, 83% over 2 steps; (c) **5**, 2,4,6-trichlorobenzoyl chloride, TEA, DMAP, THF, 30 min, -20°C, 54%.

Andrographolide as starting material for chemical synthesis of ANDRO-NBD was purchased from Bepharm Inc. (Shanghai, China), while andrographolide used in various bioactivity assays was from Sigma-Aldrich (St. Louis, MO, USA). The 3-(4,5-dimethylthiazol-2-yl)-2,5-dipheyltetrazolium bromide (MTT) was purchased from Sigma-Aldrich. Synthetic scheme of ANDRO-NBD is shown in Fig 2 and the characterization of reaction intermediates is shown in S1 Fig.

### Cells and Culture Conditions

The ts-v-Src RK3E cells were created and cultured as previously described [12]. H-1299, HT-29 and B16F10 cells (American Type Culture Collection; Manassas, USA) were obtained from Dr. Chao-Hsiung Lin, National Yang-Ming University, Taiwan). A2058, K562 and MDA-MB-231 cells were purchased from Bioresource Collection and Research Center (BCRC, Hsinchu, Taiwan). BJ-1 cells [13] were from Dr. Yeu Su at National Yang-Ming University, Taiwan. H-1299 and HT-29 cells were cultured in RPMI medium (Gibco, Grand Island, NY) containing 10% fetal bovine serum (FBS, Biological Industries, Kibbutz Beit Haemek, Israel), and 1% penicillin-streptomycin-glutamine (PSG). K562 cells were cultured in RPMI medium supplemented with 10% FBS, 1% PSG, and 1% sodium pyruvate (Gibco, Grand Island, NY). B16F10, A2058 and BJ-1 cells were cultured in DMEM medium (Gibco, Grand Island, NY) containing 10% FBS and 1% PSG. MDA-MB-231 cells were cultured in DMEM medium containing 10% FBS, 1% PSG, and 1% sodium pyruvate. All cells were incubated in a 5% humidified incubator at 35°C.

### Determination of Morphological Alterations

The ts-v-Src cells (10^5^) were seeded in MP-6 plates and grown at 35°C overnight. The cells were treated with vehicle or drugs for 24 h, and cellular morphology was photographed under a phase-contrast microscope at 200x magnification.

### Detection of Fluorescent Cellular Imaging

The ts-v-Src cells (10^5^) were seeded in MP-6 plates, grown at 35°C overnight and co-treated with ANDRO-NBD and various organelle trackers (Invitrogen, Carlsbad, CA, USA). The images were analyzed using a laser confocal microscope with processing software Laser confocal microscope (ZEISS LSM 700, Oberkochen, Germany).

### Western Blot

Vehicle- and drug-treated cells were lysed in modified RIPA buffer [50 mM Tris (pH 7.4), 150 mM NaCl, 1% Nonidet P-40, 0.25% sodium deoxycholate, 5 mM EDTA (pH 8.0), and 1 mM EGTA (pH 8.0)] containing 0.5% protease inhibitor cocktail (Sigma-Aldrich). Total protein (50 μg) was analyzed as described previously [14]. Antibody against v-Src (clone EC10) was purchased from Upstate Biotechnology (Lake Placid, NY, USA); Anti-Hsp90α and anti-Bcr antibodies antibodies were purchased from Cell Signaling (Beverly, CA, USA). Anti-GAPDH was purchased from GeneTex (Irvine, CA, USA).

### Migration Assays

B16F10 cells (3x10^4^) were suspended in serum-free medium in insert (12-mm diameter, membrane pore size 8 μm, Greiner bio-one, Frickenhausen, Germany) and the insert/cells were placed in a 24-well plate which contains DMEM medium plus 10% FBS in each well. After a 20-h incubation, all medium were removed and the insert/cells were fixed with 4% paraformaldehyde. The noninvading cells on the lower surface of inserts were cleared by cotton swabs and the insets were then stained with 2% crystal violet. For quantification of migrated cells, five independent fields at 100x magnification were photographed and the stained cells were counted.

### MTT Assays

Cell viability was examined by MTT assays as described previously [15]. Briefly, sub-confluent cells (5000~8000 cells) were seeded in 96-well plates for 24 h and treated with andrographolide or ANDRO-NBD at 0.3, 1, 3, 10, or 30 μM for 48 h. Afterwards, the cells were treated with MTT (0.5 mg/mL) for 4 h and then incubated with solubilization buffer (12% SDS, 45% DMF, pH 4.7) overnight. Cell viability was measured at 550/650 nm of absorbance by ELISA reader (Labsystems Multiskan RC, Finland). The IC_50_ was determined using GraphPad 5.0 based on MTT data from three to four independent experiments.

### Construction of His-p50 (NF-κB) Plasmid

The pCMV4-p50 plasmid was purchased from Addgene (Cambridge, MA, USA). The cDNA fragment of p50 was obtained by PCR and subcloned into the *NheI-XhoI* sites of pET-23a (+) vector to yield pET-23a (+) p50. The pET-23a (+) hnRNPK was constructed by cloning the hnRNPK cDNA to *Nhe* I and *Xho* I sites in pET-23a (+) vector.

### Expression and Purification of Recombinant Proteins

*Escherichia coli* BL21 DE3 pLysS cells were transformed with pET-23a (+) p50 or pET-23a (+) hnRNPK and grown in LB broth. When the OD_600_ reached 0.4~0.6, the bacterial culture was supplemented with IPTG (final concentration 0.4 mM). After a 3-h incubation at 30°C, the cells were harvested, resuspended in binding buffer (5 mM imidazole, 0.5 M NaCl, 20 mM Tris-HCl) and disrupted by sonication. The cell lysate was further centrifuged (10,000 rpm, 4°C, 15 min) to collect supernatant which was then incubated with Ni-NTA agarose beads (Quagen, Chatsworth, CA, USA). The recombinant protein was then purified using a procedure described by the manufacturer. The eluted fractions were subsequently dialyzed to remove imidazole. The proteins were finally dissolved in PBS.

### Reporter Assays

The RAW 264.7/Luc-P1 cells (4x10^5^ cells in 24-well plates) were treated with andrographolide, ANDRO-NBD or vehicle (0.1% DMSO) for 1 h and then LPS for 6 hours, collected, and analyzed using luciferase assays (Promega, Madison, WI) as described previously [16]. The luminescence was measured with an Infinite^®^ 200 PRO (Tecan Group Ltd, Männedorf, Switzerland).

### In Vitro Binding Assay

One microgram of recombinant protein was incubated with ANDRO-NBD in PBS buffer for 1 h at 25°C. The samples were then analyzed on 10% SDS-polyacrylamide gels. To detect the fluorescent signals, the gels were scanned (excitation, 488 nm; emission 520 nm) by Typhoon TRIO scanner (GE Healthcare).

### Statistics

The data are expressed as the mean ± SD of three independent experiments. A *p*-value of < 0.05 indicated a statistically significant difference as determined by Student’s t-test.

## Results

### Chemical Synthesis of ANDRO-NBD

A previous structure-activity-relationship study suggested that andrographolide derivatives with a bulky, hydrophobic group at C-19 position and an acetyl group at C-14 position exhibited higher cytotoxic activities against cancer cells than andrographolide [17]. We therefore designed a fluorescent andrographolide derivative carrying an acetoxy group at C-14 and a nitrobenzoxadiazole (NBD) fluorophore linked via an ester bond to the C-19 hydroxyl group (ANDRO-NBD in Fig 1). The NBD moiety is a hydrophobic fluorophore which provides cell permeability for potential *in vivo* applications [18, 19].

Synthesis of ANDRO-NBD began with protection of the C-3/C-19 diol of andrographolide through acetonide formation, as shown in Fig 2. Upon treatment of andrographolide with 2,2-dimethoxypropane and a catalytic amount of pyridinium *p*-toluenesulfonate (PPTS) in acetone, acetonide **3** was obtained in high yield (97%). The C-14 hydroxyl group of compound **3** was then subjected to acetylation, followed by deprotection of the acetonide group, to give compound **4** in good yield (83%) over two steps. It is interesting to note that compound **4**, a C-14 acetyl derivative of andrographolide **1**, is soluble in CH_2_Cl_2_. In contrast, andrographolide **1** is relatively insoluble in CH_2_Cl_2_. The largely increased solubility of compound **4** in organic solvents also helped promote its derivatization in the following step. The final and most critical step for the preparation of compound **2** is to attach NBD-containing reagent **5** [20] to the C-19 hydroxyl group of compound **4** through ester bond formation. Initial attempts using acyl chloride or DCC/DMAP coupling conditions failed to give the desired product **2**. Instead, elimination of the C-14 acetoxyl group had occurred. It was later found that Yamaguchi esterification protocol (2,4,6-trichlorobenzoyl chloride/TEA/DMAP/THF) [21] could successfully give ANDRO-NBD in moderate yield (54%).

### ANDRO-NBD Showed Comparable Anticancer Profiles to Andrographolide

**Fig 3:**
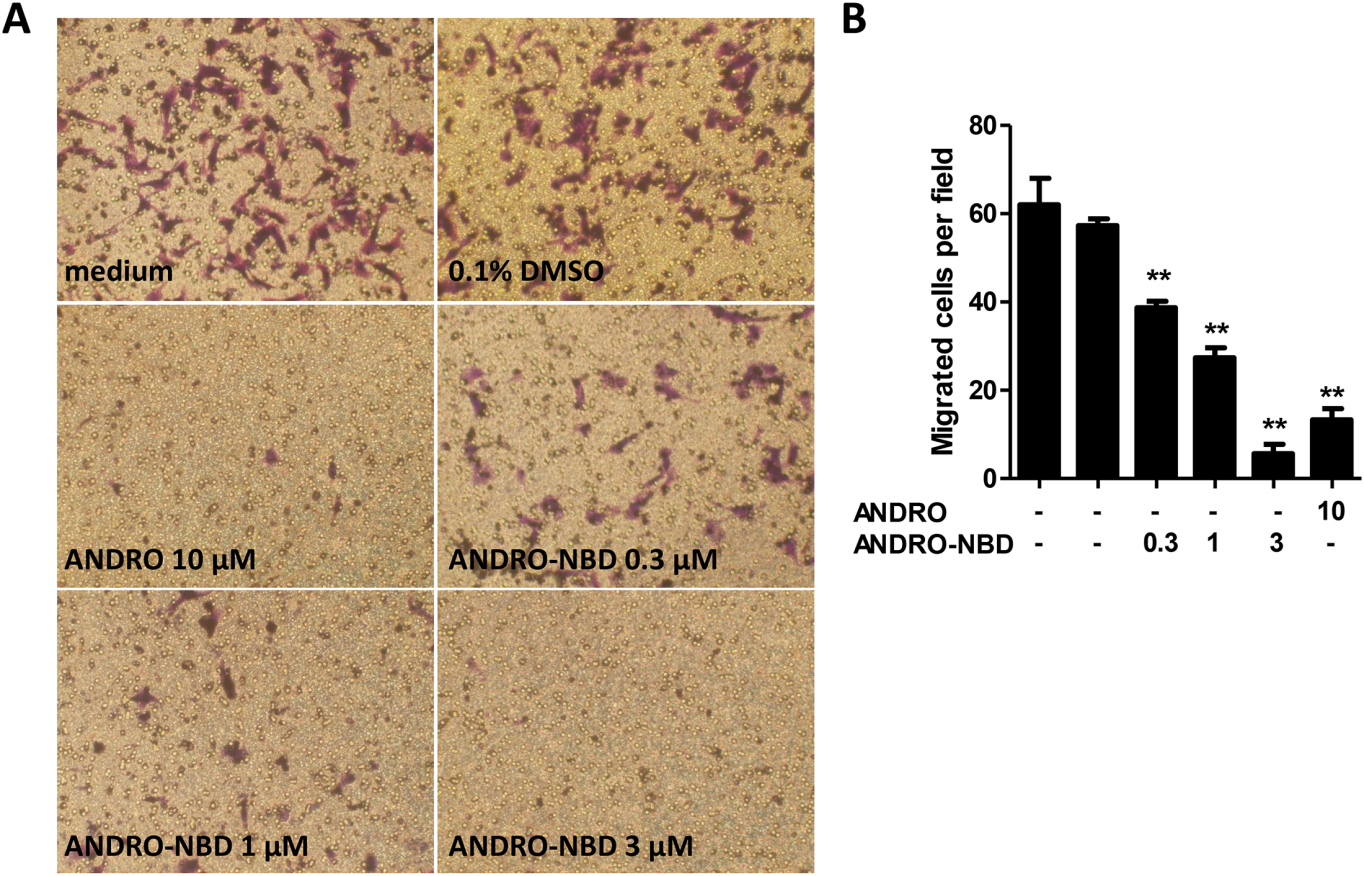
ANDRO-NBD suppressed migration activity of melanoma cells. B16F10 cells (3x10^4^) were treated with vehicle (0.1% DMSO), andrographolide (ANDRO), or indicated concentrations of ANDRO-NBD and analyzed using migration assays. (A) The migrated cells were stained with 2% crystal violet and photographed under a phase-contrast microscope (100x). (B) Quantification of the migrated cells. The stained cells from five independent fields (100x) were photographed and counted. Data are presented as mean ± SD, and ** indicates significant difference *versus* control (p<0.01).

We first examined whether ANDRO-NBD retained the bioactivities of andrographolide. To this end, the anticancer activities of ANDRO-NBD were measured at different aspects. As shown in Table 1, ANDRO-NBD showed potent cytotoxic effects on various human cancer cell lines. Notably, the cytotoxic effects of ANDRO-NBD on all cancer cell types were more potent than andrographolide. Furthermore, ANDRO-NBD significantly suppressed the migration activity of highly metastatic melanoma cells B16F10 at a lower concentration than andrographolide (Fig 3).

**Table 1:** Cytotoxicity of ANDRO-NBD on various human cell lines.

Cell line	Cell type	IC_50_ (μM)	
		andrographolide	ANDRO-NBD
MDA-MB-231	Breast cancer	>30	9.83±1.13
K562	Leukemia	24.12±2.91	15.28±0.94
A2058	melanoma	26.10±5.96	4.52±0.46
H-1299	Lung cancer	22.55±4.3	7.39±1.96
HT-29	Colon cancer	>30	12.24±2.18
BJ-1	Immortalized fibroblast	>30	19.01±1.79

**Fig 4:**
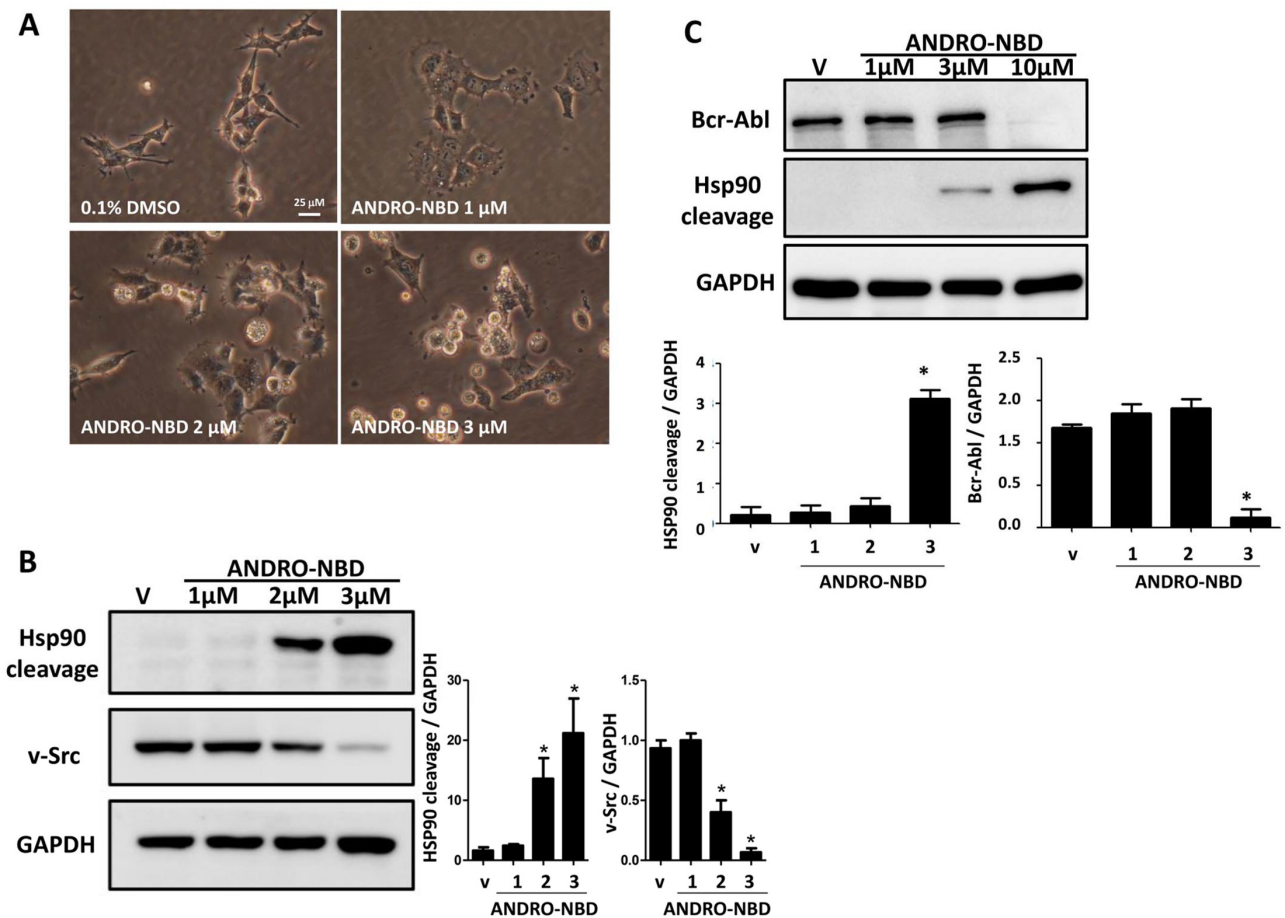
ANDRO-NBD suppressed v-Src-mediated cellular transformation and reduced the expression of oncoproteins, v-Src and Bcr-abl. (A) The ts-v-Src cells (10^5^) were treated with vehicle or ANDRO-NBD for 24 h at 35°C and cell morphology was photographed under a phase-contrast microscope. (B) Vehicle- and ANDRO-NBD treated ts-v-Src cells grown at 35°C were lysed in modified RIPA buffer and analyzed with Western blot. (C) Vehicle- and ANDRO-NBD-treated K562 cells were lysed in modified RIPA buffer and analyzed with Western blot. Data are presented as mean ± SD, and * indicates significant difference *versus* vehicle (*p<0.05).

Our previous study showed that andrographolide could impair malignant cancer phenotypes through induction of Hsp90 cleavage as well as degradation of Hsp90 client oncoproteins (v-Src and Bcr-abl) [14]. We herein demonstrated that ANDRO-NBD also significantly suppressed v-Src-mediated cell transformation, caused Hsp90 cleavage, and reduced v-Src expression (Fig 4). Notably, the effective concentrations of ANDRO-NBD in aforementioned experiments were lower than those of andrographolide reported previously [14]. Together, ANDRO-NBD exhibited an anticancer spectrum comparable to its parental compound, andrographolide.

### Cellular Uptake and Subcellular Location of ANDRO-NBD

**Fig 5:**
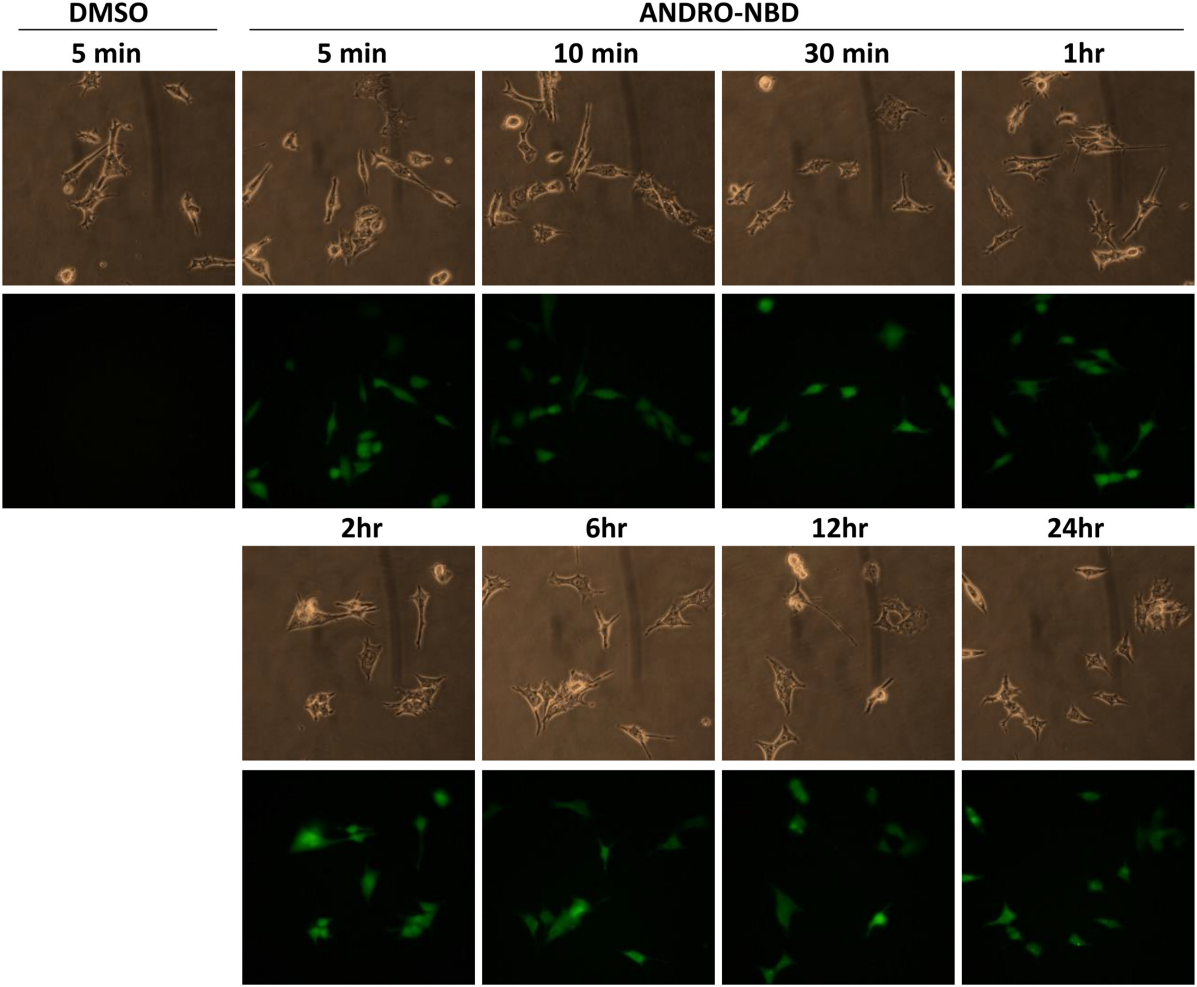
The kinetics of ANDRO-NBD uptake by v-Src-transformed cells. The ts-v-Src cells (2x10^5^) grown at 35°C were treated with ANDRO-NBD (2 μM) for the indicated time periods. The images were photographed using a phase-contrast microscope with or without fluorescence illuminator.

The uptake kinetics and distribution of ANDRO-NBD in cancer cells were further examined. Upon ANDRO-NBD treatment, the fluorescent signals could be detected inside the ts-v-Src cells within five minutes and the intensity of fluorescence increased as incubation was prolonged. Moreover, such fluorescent signals retained after 24-h incubation (Fig 5). In addition, similar kinetics of ANDRO-NBD uptake was observed in breast cancer cell MDA-MB-231 (S2 Fig).

**Fig 6:**
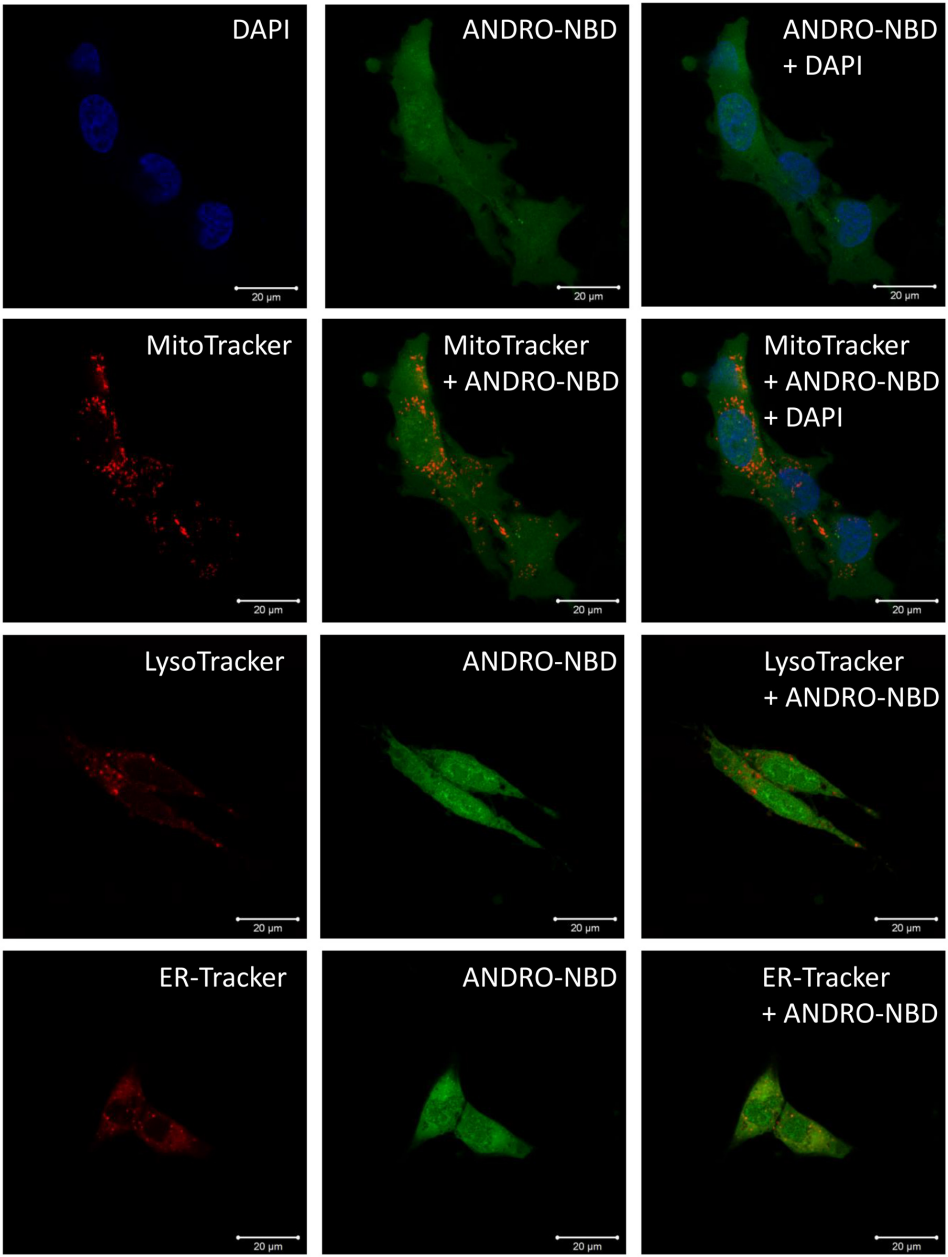
Cellular distribution of ANDRO-NBD in v-Src-transformed cells. The ts-v-Src cells (10^5^) grown at 35°C were co-treated with ANDRO-NBD (2 μM) and the indicated organelle tracker for 2 hours. DAPI, MitoTracker, LysoTracker and ERTracker are for staining nucleus, mitochondria, lysosome and endoplasmic reticulum (ER), respectively. The images were analyzed using a Laser confocal microscope (ZEISS LSM 700).

The subcellular distribution of ANDRO-NBD was also investigated. The ts-v-Src cells after ANDRO-NBD treatment were stained with various organelle trackers and DAPI. As shown in Fig 6, the fluorescent signal of ANDRO-NBD could be detected in nucleus, cytoplasm, mitochondria, endoplasmic reticulum (ER) and lysosomes. We have confirmed that NBD alone did not cause background fluorescence in cell culture (S3 Fig). Furthermore, pretreatment of andrographolide significantly reduced the ANDR-NBD-mediated fluorescence, indicating andrographolide and ANDRO-NBD share overlapping localization within cells (S3 Fig).

### ANDRO-NBD Covalently Bound to Target Proteins of Andrographolide

**Fig 7:**
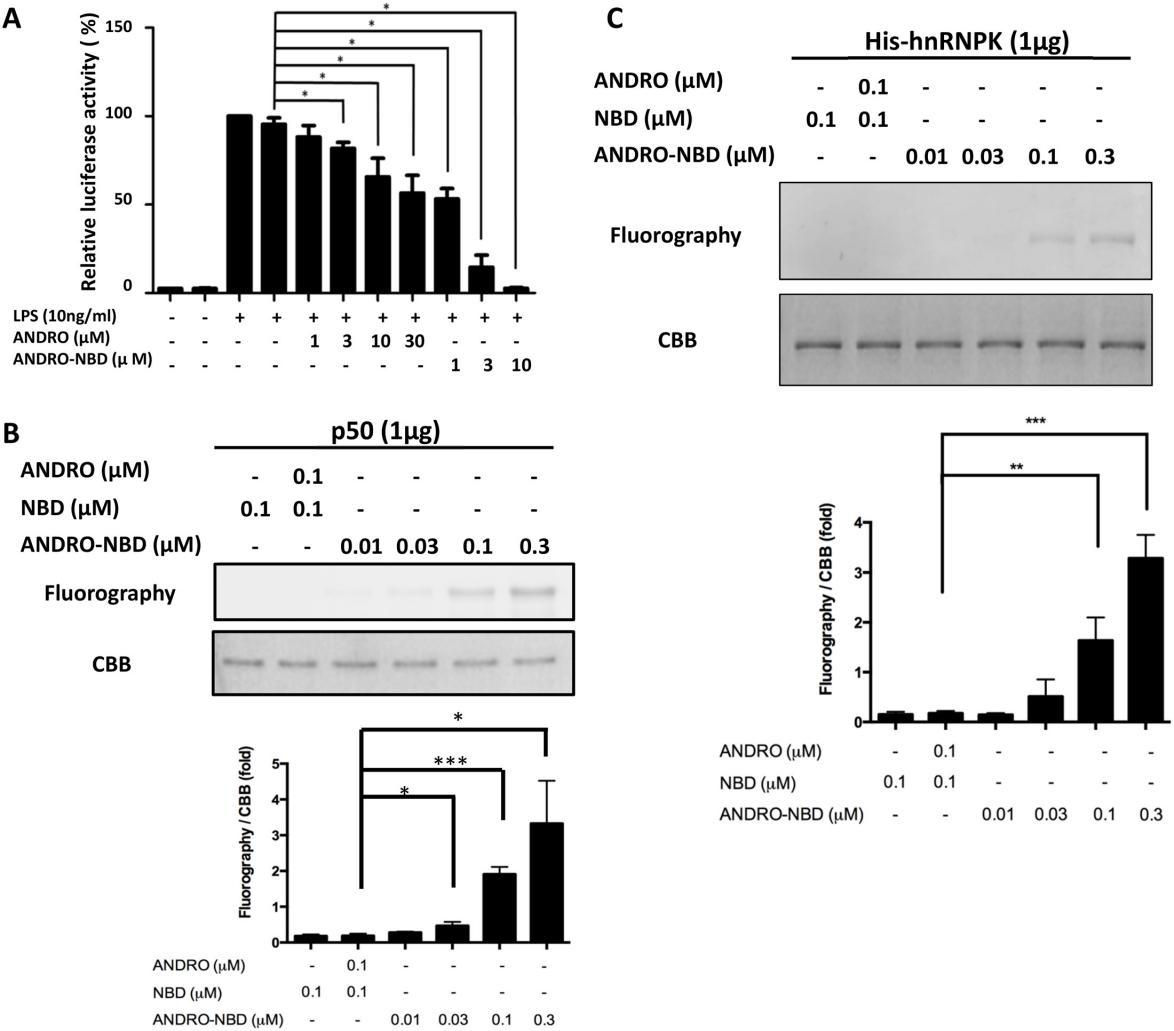
ANDRO-NBD suppresses transcriptional activation of NF-κB and could covalently bind to known target proteins of andrographolide. (A) The RAW 264.7/Luc-P1 cells (4x10^5^) were treated with vehicle (0.1% DMSO) or indicated compounds, and analyzed using reporter assays. In both (B) and (C), one microgram of recombinant protein was incubated with ANDRO-NBD in PBS buffer for 1 h at 25°C, and then analyzed with 10% SDS-polyacrylamide gels. The fluorescent signals were detected using Typhoon TRIO scanner. Quantitation data from three independent experiments are presented as mean ± SD, and * indicates significant difference *versus* control (p<0.05).

Andrographolide has been previously shown to inhibit the DNA-binding activity of NF-κB through forming a covalent bond with its p50 subunit [8]. Likewise, ANDRO-NBD could suppress the LPS-mediated transcriptional activation of NF-κB (Fig 7A) and covalently label p50 subunit with fluorescent signal in an *in vitro* labeling assay (Fig 7B). Taking advantage of the fluorescent tag of ANDRO-NBD, it could be further applied to identify other possible target proteins of andrographolide. A list of candidate andrographolide-bound proteins, including hnRNPK, was previously proposed using a click chemistry-based proteomics approach [9]. We herein demonstrated that ANDRO-NBD could be covalently bound to hnRNPK through *in vitro* labeling experiment (Fig 7C), providing direct evidence that hnRNPK is an authentic target of andrographolide.

## Discussion

In this study, we demonstrated that ANDRO-NBD shows a comparable anticancer profile to andrographolide (Table 1, Figs 3 and 4) and could covalently label the target proteins (NF-κB and hnRNPK) of andrographolide with fluorescence (Fig 7). Accordingly, ANDRO-NBD is considered as a fluorescent bioactive surrogate of andrographolide and provides an effective tool for andrographolide-related research.

In the spectrum of anticancer assays we have examined, ANDRO-NBD exhibited superior anticancer activity to andrographolide (Table 1, Figs 3 and 4). Our data is in accordance with previous studies that addition of a hydrophobic group at C-19 position and an acetyl group at C-14 position in chemical structure of andrographolide significantly improved its cytotoxic activity against cancer cells [17, 22]. It is implicated that the hydrophobicity of NBD moiety enhances cell permeability [18, 19] and therefore contributes to the effective accessibility of ANDRO-NBD to target proteins, as shown in Fig 5. In addition, whether ANDRO-NBD also possesses andrographolide-like therapeutic potency in other diseases certainly merits further investigation.

Fluorescence imaging is an efficient technique for real-time tracking of intracellular molecules. As shown in Figs 5 and 6, ANDRO-NBD was ubiquitously distributed in nucleus, cytoplasm, mitochondria, lysosome, and ER, suggesting its effective accessibility to most of the organelles. Interestingly, previously reported biological activities of andrographolide, such as transcriptional regulation, apoptosis, autophagy, and cell cycle regulation, indeed are associated with aforementioned cell locations [2, 4]. Furthermore, protein targets of andrographolide proposed by different studies also imply the ubiquitous distribution of andrographolide-bound proteins [9], [11]. Therefore, it is speculated that andrographolide, like ANDRO-NBD, also exhibits its activities at a variety of locations in cells.

A few andrographolide-based probes have been previously reported to study the action mechanism of andrographolide. The probe described by Wang et al. allows click chemistry-based protein profiling and target identification of andrographolide [9]. Another probe was reported for both *in situ* imaging and target identification, which carries a capped fluorophore at C-14 and can release fluorescent signal upon nucleophilic attack by the corresponding targets [11]. However, it should be noted that such fluorescent signals were not retained on the labeled targets. In contrast, ANDRO-NBD described in the present study can directly form covalent bonds with target proteins of andrographolide and thus establish a fluorescent tag on these proteins for the following fluorescent detection. Moreover, cellular ANDRO-NBD-labeled proteins could be further enriched with a NBD-specific antibody and subsequently isolated for protein identification.

## Conclusions

In this study, we described the synthesis and anticancer activity of a fluorescent andrographolide derivative ANDRO-NBD which was also demonstrated to be an andrographolide-mimic probe to analyze the uptake kinetics, cellular distribution and cellular targets of andrographolide. In the future, this probe can be applied to identify the therapeutically relevant molecular targets underlying pharmacological mechanism of andrographolide in various diseases. Based on the superior inhibitory activity of ANDRO-NBD to andrographolide, the therapeutic potential of ANDRO-NBD in other diseases also warrants further evaluation.

## Supporting Information

S1 Fig Characterization of ANDRO-NBD and reaction intermediates described in Fig 2. (PDF)

S2 Fig The uptake kinetics of ANDRO-NBD in MDA-MB-231. (PDF)

S3 Fig Pretreatment of andrographolide significantly reduced ANDRO-NBD- mediated fluorescence. (PDF)
