# Supplementary material for: Development of a Bifunctional Andrographolide-Based Chemical Probe for Pharmacological Study
Source: PLoS One. 2016 Apr 1;11(4):e0152770. doi: 10.1371/journal.pone.0152770 (PMC4818061; doi:10.1371/journal.pone.0152770)

S1 Fig. Characterization of ANDRO-NBD and reaction intermediates described in Fig 2.

(*S*,*E*)-4-Hydroxy-3-(2-((4a*R*,6a*S*,7*R*,10a*S*,10b*R*)-3,3,6a,10b-tetramethyl-8-methylenedecahydro-1H-naphtho[2,1-*d*][1,3]dioxin-7-yl)ethylidene)dihydrofuran-2(3*H*)-one (3). To a stirred solution of andrographolide **1** (5.0 g, 14 mmol) in 285 mL acetone was added 2,2-dimethoxypropane (16 mL, 0.13 mol), followed by addition of a catalytic amount of PPTS (179 mg, 0.714 mmol). After stirring for 30 min, the solvent was evaporated to dryness and EtOAc (300 mL) was added. It was washed with saturated $NaHCO_3$ (100 mL x3), H_2O (100 mL x2), and brine (100 mL). The organic layer was dried over Na_2SO_4, filtered, and evaporated under vacuum to afford the crude solid product. The solid was washed with hexane, collected by filtration to provide **3** (5.4 g, 97%) as a white solid, mp 194-195 °C. R_f = 0.53 (hexane/EtOAc = 9/1). ^{1}H NMR (400 MHz, $CDCl_3$): δ 6.88 (t, 1H, *J* = 6.8 Hz), 4.97 (m, 1H), 4.86 (s, 1H), 4.59 (s, 1H), 4.40 (dd, 1H, *J* = 10.4, 6.1 Hz), 4.20 (dd, 1H, *J* = 10.4, 1.8 Hz), 3.92 (d, 1H, *J* = 11.6 Hz), 3.44 (m, 1H), 3.31 (m, 1H), 3.13 (d, 1H, *J* = 1.6 Hz), 2.53 (m, 2H), 2.37 (m, 1H), 1.99–1.89 (m, 2H), 1.81–1.67 (m, 4H), 1.37 (s, 3H), 1.32 (s, 3H), 1.28–1.19 (m, 3H), 1.15 (s, 3H). ^{13}C NMR (100 MHz, $CDCl_3$): δ 170.4 (C), 149.0 (CH), 146.9 (C), 127.9 (C), 108.9 (CH_2), 99.1 (C), 76.3 (CH), 74.5 (CH_2), 65.9 (CH), 63.8 (CH_2), 55.9 (CH), 52.2 (CH), 38.3 (C), 37.8 (C), 37.6 (CH_2), 34.5 (CH_2), 27.0 (CH_3), 26.1 (CH_3), 25.3 (CH_3), 24.9 (CH_2), 24.9 (CH_2), 23.1 (CH_2), 16.1(CH_3).□IR (KBr): 3473, 2939, 1745, 1685, 1222, 1066 cm^{-1}. HRMS calcd for $C_{46}H_{68}NaO_{10}$ $(2M+Na)^+$ 803.4705, found 803.4744.

(*S*,*E*)-4-(2-((1*R*,4a*S*,5*R*,6*R*,8a*S*)-6-Hydroxy-5-(hydroxymethyl)-5,8a-dimethyl-2-methylenedecahydronaphthalen-1-yl)ethylidene)-5-oxotetrahydrofuran-3-yl

acetate (4). A solution of **3** (3.0 g, 7.7 mmol) in 25 mL of acetic anhydride was heated to reflux for 1.5 h. The reaction mixture was then allowed to cool to room temperature, diluted with EtOAc (200 mL), and extracted with saturated $NaHCO_3$ (80 mL x 3), H_2O (80 mL x 2), brine (80 mL). The organic layer was dried over Na_2SO_4, filtered, and evaporated. The crude product was dissolved in 30 mL of AcOH/H_2O (7/3) solution and stirred for 10 min. EtOAc (200 mL) was added and washed consecutively with saturated $NaHCO_3$ (80 mL x 3), H_2O (80 mL x 2), brine (80 mL). The organic layer was dried over Na_2SO_4, filtered, and evaporated. The residue was subjected to silica gel column chromatography eluted with CH_2Cl_2/MeOH (98/2) and the product was further recrystallized from CH_2Cl_2/ether. The desired product **4** was obtained as a white crystal (2.4 g, 83%), mp 165-167 ^{o}C. R_f = 0.5 (chloroform/MeOH = 9/1). ^{1}H NMR (400 MHz, $CDCl_3$): δ 6.93 (m, 1H), 5.85 (d, 1H, *J* = 6.0 Hz), 4.81 (s, 1H), 4.48 (dd, 1H, *J* = 11.2, 6.1 Hz), 4.43 (s, 1H), 4.18 (dd, 1H, *J* = 11.2, 1.8 Hz), 4.10 (d, 1H, *J* = 10.4 Hz), 4.36 (d, 1H, *J* = 4.4 Hz), 3.46 (dd, 1H, *J* = 8.2, 1.3 Hz), 3.38 (m, 1H), 3.23 (dd, 1H, *J* = 10.4, 8.2 Hz), 2.43–2.26 (m, 3H), 2.05 (s, 3H), 1.90 (m, 1H), 1.78–1.72 (m, 4H), 1.66 (m, 1H) 1.27–1.10 (m, 6H), 0.60 (s, 3H). ^{13}C NMR (100 MHz, $CDCl_3$): δ 170.4 (C), 169.1 (C), 150.5 (CH), 146.7 (C), 123.7 (C), 108.5 (CH_2), 80.0 (CH), 71.5 (CH_2), 67.6 (CH), 64.0 (CH_2), 55.7 (CH), 55.0 (CH), 42.6 (C), 38.7 (C), 37.6 (CH_2), 36.8 (CH_2), 27.9 (CH_2), 25.2 (CH_2), 23.5 (CH_2), 22.7 (CH_3), 20.6 (CH_3), 15.0 (CH_3). IR (KBr): 3410, 2937, 1739, 1676, 1371, 1229, 1022 cm^{-1}. HRMS calcd for $C_{22}H_{32}NaO_6$ $(M+H)^+$ 415.2091, found 415.2092.

((1*R*,2*R*,4a*S*,5*R*,8a*S*)-5-((*E*)-2-((*S*)-4-Acetoxy-2-oxodihydrofuran-3(2*H*)-ylidene)ethyl)-2-hydroxy-1,4a-dimethyl-6-methylenedecahydronaphthalen-1-yl)methyl 2-(methyl(7-nitrobenzo[c][1,2,5]oxadiazol-4-yl)amino)acetate (2, ANDRO-NBD). To a solution of **5** (167.1 mg, 0.663 mmol), 2,4,6-trichlorobenzoyl chloride (103.6 μL,

0.663 mmol), TEA (92 μL, 0.663 mmol) in 10 mL of anhydrous THF was stirred for 5 min in an ice/salt bath (-16 ^{o}C) under argon in the dark, followed by adding **4** (200.0 mg, 0.510 mmol) and DMAP (81.0 mg, 0.663 mmol). After stirring for 30 min, the reaction mixture was quenched by adding H_2O (1 mL) and was diluted with CH_2Cl_2 (60 mL). It was washed with 5% $NaHCO_3$ (30 mL x3), 5% citric acid (30 mL x 2), H_2O (30 mL x2), and brine (30 mL). The organic layer was dried over Na_2SO_4, filtered, and evaporated. The residue was purified with silica gel column chromatography eluted with CH_2Cl_2/EtOAc (6/4). The fractions containing **2** were pooled and evaporated. Compound **2** was obtained by reprecipitation with CH_2Cl_2/ether as an orange solid (172.1 mg, 54%), mp 110-112 ^{o}C. R_f = 0.35 (chloroform/EtOAc = 3/7). ^{1}H NMR (400 MHz, $CDCl_3$): δ 8.46 (d, 1H, *J* = 8.8 Hz), 6.95 (m, 1H), 6.20 (d, 1H, *J* = 8.8 Hz), 5.88 (d, 1H, *J* = 6.0 Hz), 4.94–4.79 (m, 3H), 4.52 (dd, 1H, *J* = 11.3, 6.2 Hz), 4.49–4.46 (m, 2H), 4.21 (dd, 1H, *J* = 11.2, 1.9 Hz), 4.12 (d, 1H, *J* = 11.7 Hz), 3.43 (s, 3H), 3.31 (dd, 1H, *J* = 11.6, 3.7 Hz), 2.45–2.27 (m, 3H), 2.11 (s, 3H), 1.90 (m, 1H), 1.80–1.61 (m, 5H), 1.37–1.17 (m, 4H), 1.07 (s, 3H), 0.61 (s, 3H). ^{13}C NMR (100 MHz, $CDCl_3$): δ 170.4 (C), 169.0 (C), 168.2 (C), 150.2 (CH), 146.3 (C), 145.4 (C), 144.6 (C), 144.3 (C), 135.2 (CH), 123.8 (C), 123.5 (C), 108.7 (CH_2), 102.2 (CH), 78.0 (CH), 71.5 (CH_2), 67.6 (CH), 66.4 (CH_2), 57.2 (CH_2), 55.6 (CH), 54.7 (CH), 42.2 (C), 42.2 (CH_3), 38.8 (C), 37.6 (CH_2), 36.9 (CH_2), 27.5 (CH_2), 25.0 (CH_2), 24.3 (CH_2), 22.3 (CH_3), 20.6 (CH_3), 14.3 (CH_3). IR (KBr): 2936, 1738, 1556, 1298, 1221, 1092 cm^{-1}. HRMS calcd for $C_{31}H_{38}N_4NaO_{10}$ $(M + Na)^+$ 649.2480, found 649.2485.

The ^{1}H/^{13}C NMR spectra for compounds **2**, **3**, and **4** were included in the following pages.

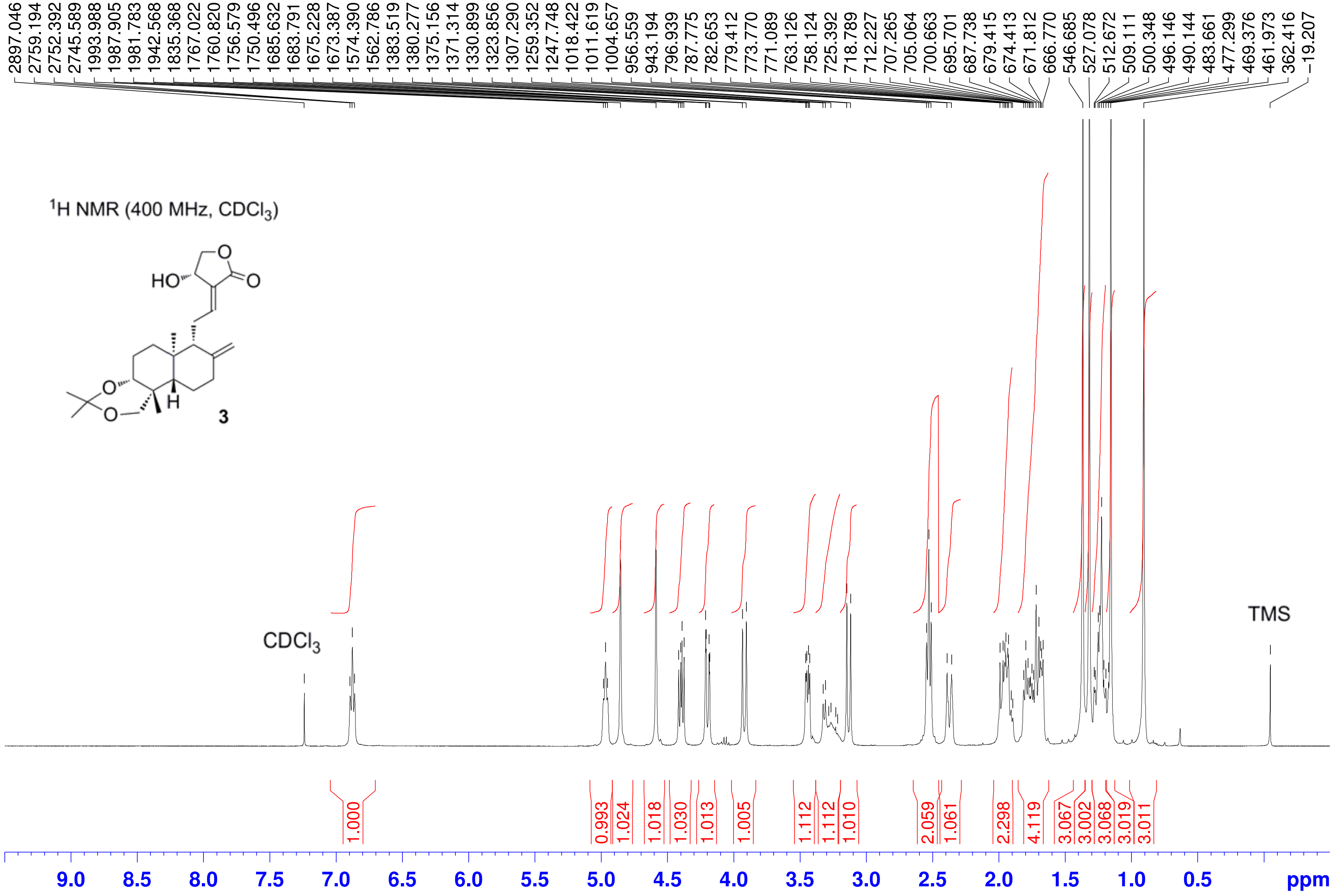

^{1}H NMR (400 MHz, CDCl$_3$)
HO
O
O
O
O
H
3
CDCl$_3$
TMS
2897.046
2759.194
2752.392
2745.589
1993.988
1987.905
1981.783
1942.568
1835.368
1767.022
1760.820
1756.579
1750.496
1685.632
1683.791
1675.228
1673.387
1574.390
1562.786
1383.519
1380.277
1375.156
1371.314
1330.899
1323.856
1307.290
1259.352
1247.748
1018.422
1011.619
1004.657
956.559
943.194
796.939
787.775
782.653
779.412
773.770
771.089
763.126
758.124
725.392
718.789
712.227
707.265
705.064
700.663
695.701
687.738
679.415
674.413
671.812
666.770
546.685
527.078
512.672
509.111
500.348
496.146
490.144
483.661
477.299
469.376
461.973
362.416
−19.207
1.000
0.993
1.024
1.018
1.030
1.013
1.005
1.112
1.112
1.010
2.059
1.061
2.298
4.119
3.067
3.002
3.068
3.019
3.011
9.0
8.5
8.0
7.5
7.0
6.5
6.0
5.5
5.0
4.5
4.0
3.5
3.0
2.5
2.0
1.5
1.0
0.5
ppm

^{13}C NMR (100 MHz, $CDCl_3$)

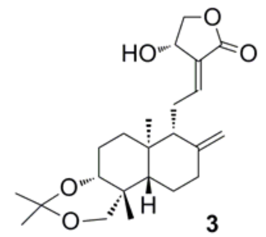

3

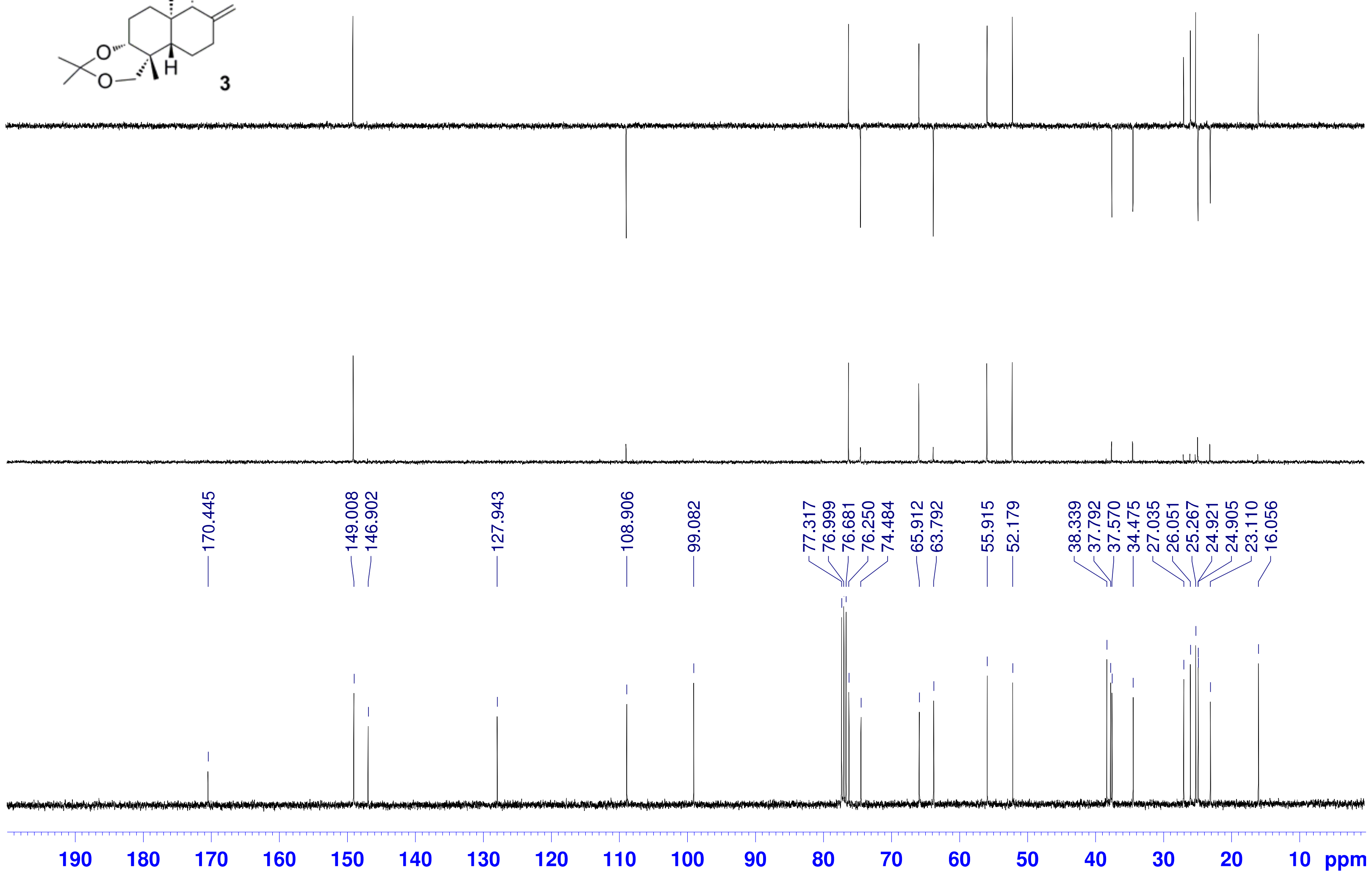

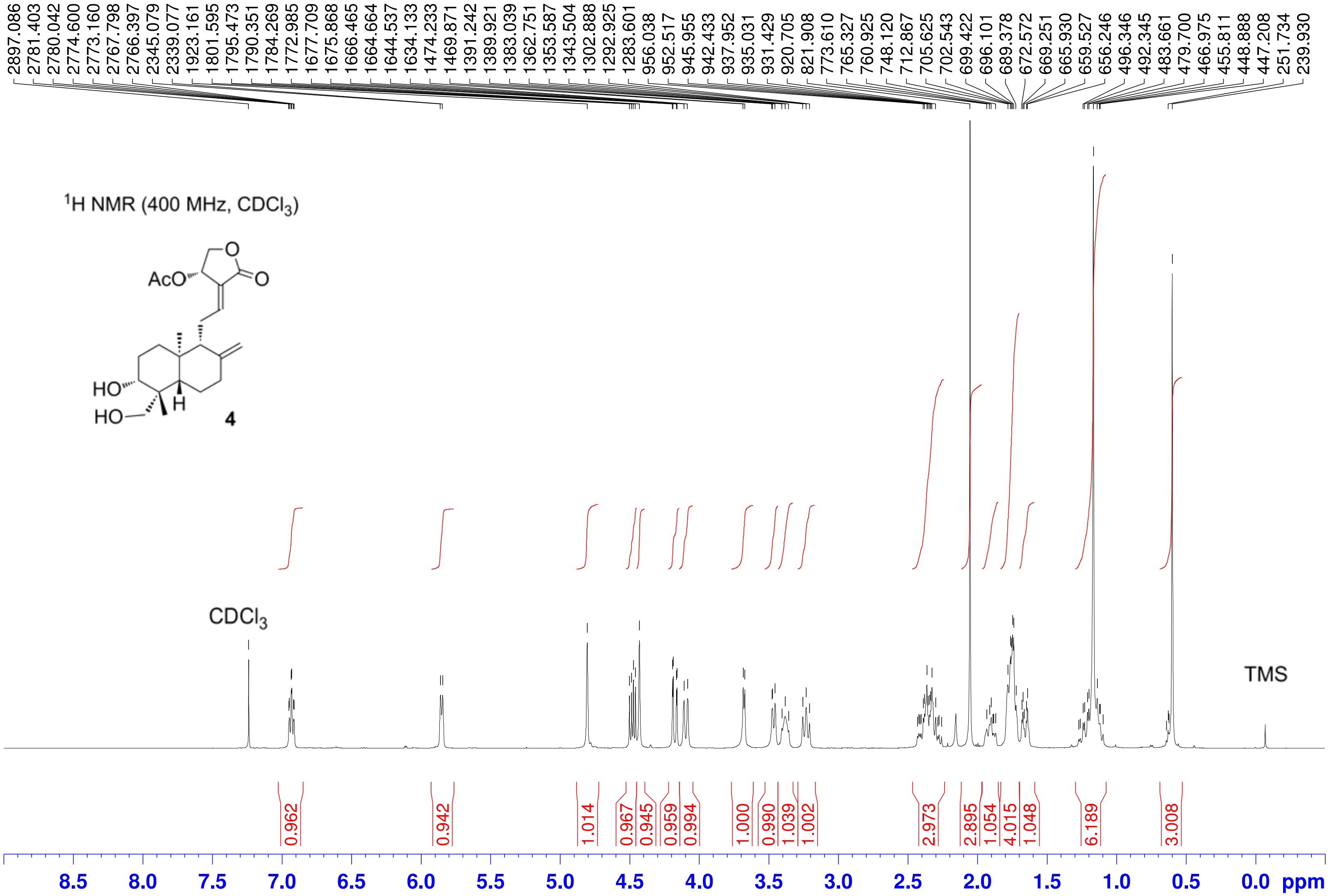

^{1}H NMR (400 MHz, $CDCl_3$)
AcO
O
O
HO
HO
H
4
$CDCl_3$
TMS
2897.086
2781.403
2780.042
2774.600
2773.160
2767.798
2766.397
2345.079
2339.077
1923.161
1801.595
1795.473
1790.351
1784.269
1772.985
1677.709
1675.868
1666.465
1664.664
1644.537
1634.133
1474.233
1469.871
1391.242
1389.921
1383.039
1362.751
1353.587
1343.504
1302.888
1292.925
1283.601
956.038
952.517
945.955
942.433
937.952
935.031
931.429
920.705
821.908
773.610
765.327
760.925
748.120
712.867
705.625
702.543
699.422
696.101
689.378
672.572
669.251
665.930
659.527
656.246
496.346
492.345
483.661
479.700
466.975
455.811
448.888
447.208
251.734
239.930
0.962
0.942
1.014
0.967
0.945
0.959
0.994
1.000
0.990
1.039
1.002
2.973
2.895
1.054
4.015
1.048
6.189
3.008
8.5
8.0
7.5
7.0
6.5
6.0
5.5
5.0
4.5
4.0
3.5
3.0
2.5
2.0
1.5
1.0
0.5
0.0
ppm

^{13}C NMR (100 MHz, $CDCl_3$)

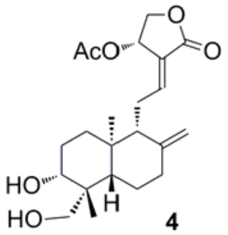

4

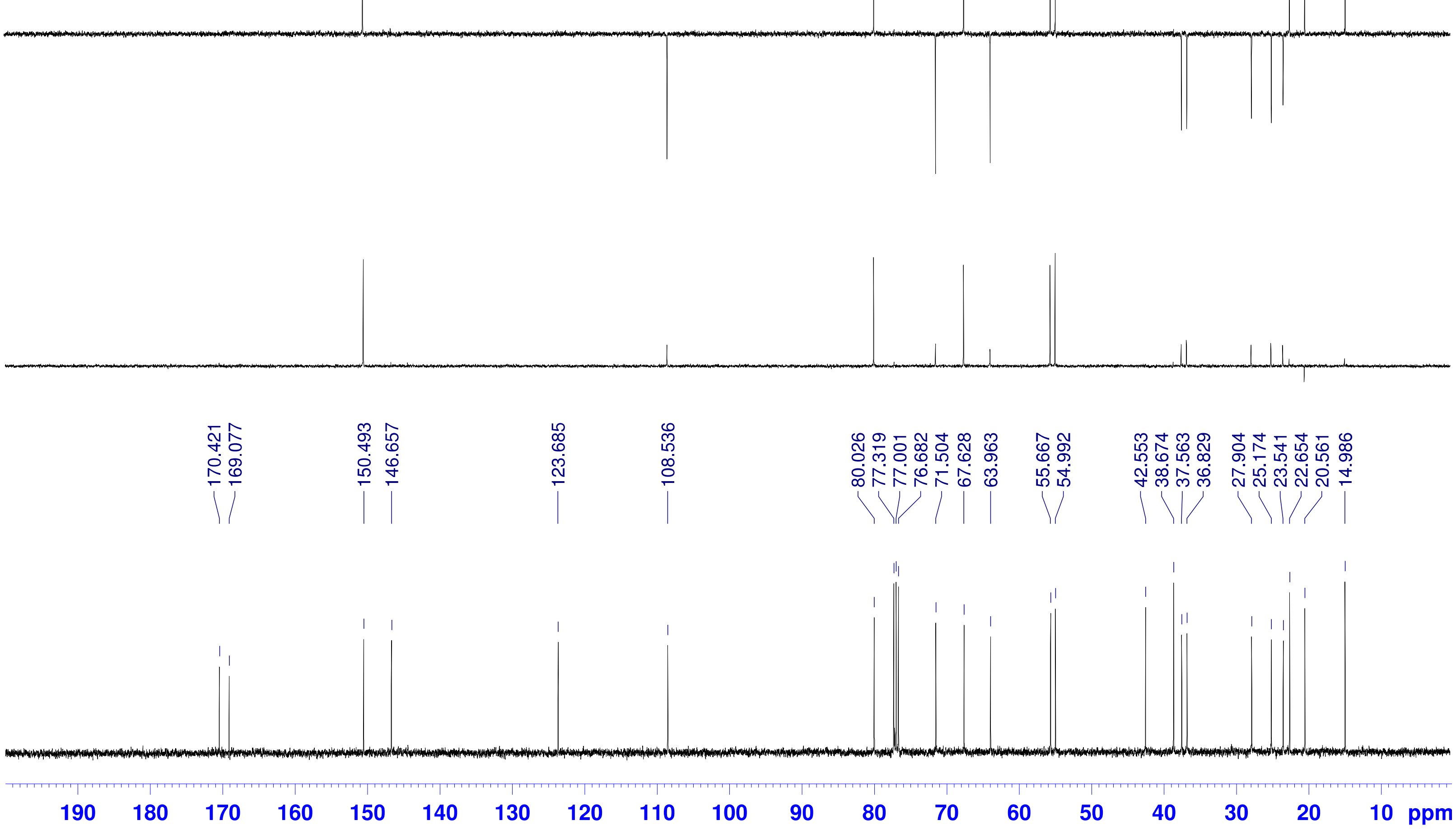

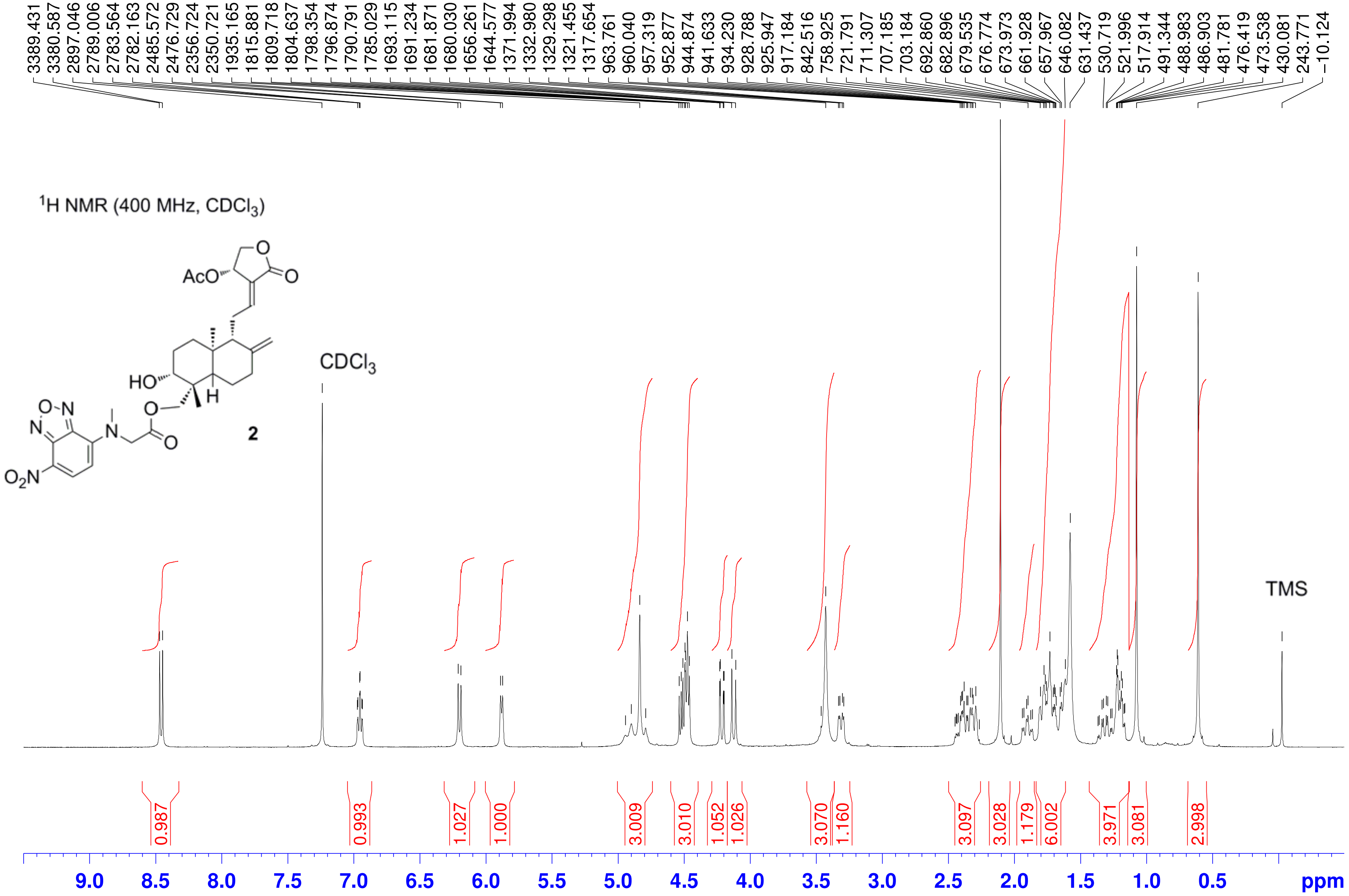

¹H NMR (400 MHz, CDCl3)
AcO
O
O
HO
H
O
O
N
O-N
N
O2N
2
CDCl3
TMS
3389.431
3380.587
2897.046
2789.006
2783.564
2782.163
2485.572
2476.729
2356.724
2350.721
1935.165
1815.881
1809.718
1804.637
1798.354
1796.874
1790.791
1785.029
1693.115
1691.234
1681.871
1680.030
1656.261
1644.577
1371.994
1332.980
1329.298
1321.455
1317.654
963.761
960.040
957.319
952.877
944.874
941.633
934.230
928.788
925.947
917.184
842.516
758.925
721.791
711.307
707.185
703.184
692.860
682.896
679.535
676.774
673.973
661.928
657.967
646.082
631.437
530.719
521.996
517.914
491.344
488.983
486.903
481.781
476.419
473.538
430.081
243.771
−10.124
0.987
0.993
1.027
1.000
3.009
3.010
1.052
1.026
3.070
1.160
3.097
3.028
1.179
6.002
3.971
3.081
2.998
9.0
8.5
8.0
7.5
7.0
6.5
6.0
5.5
5.0
4.5
4.0
3.5
3.0
2.5
2.0
1.5
1.0
0.5
ppm

^{13}C NMR (100 MHz, $CDCl_3$)

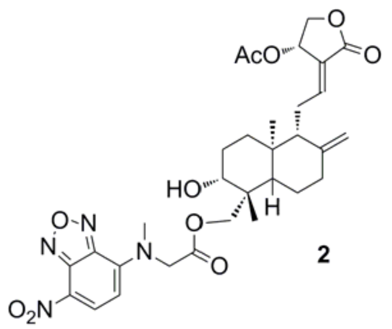

2

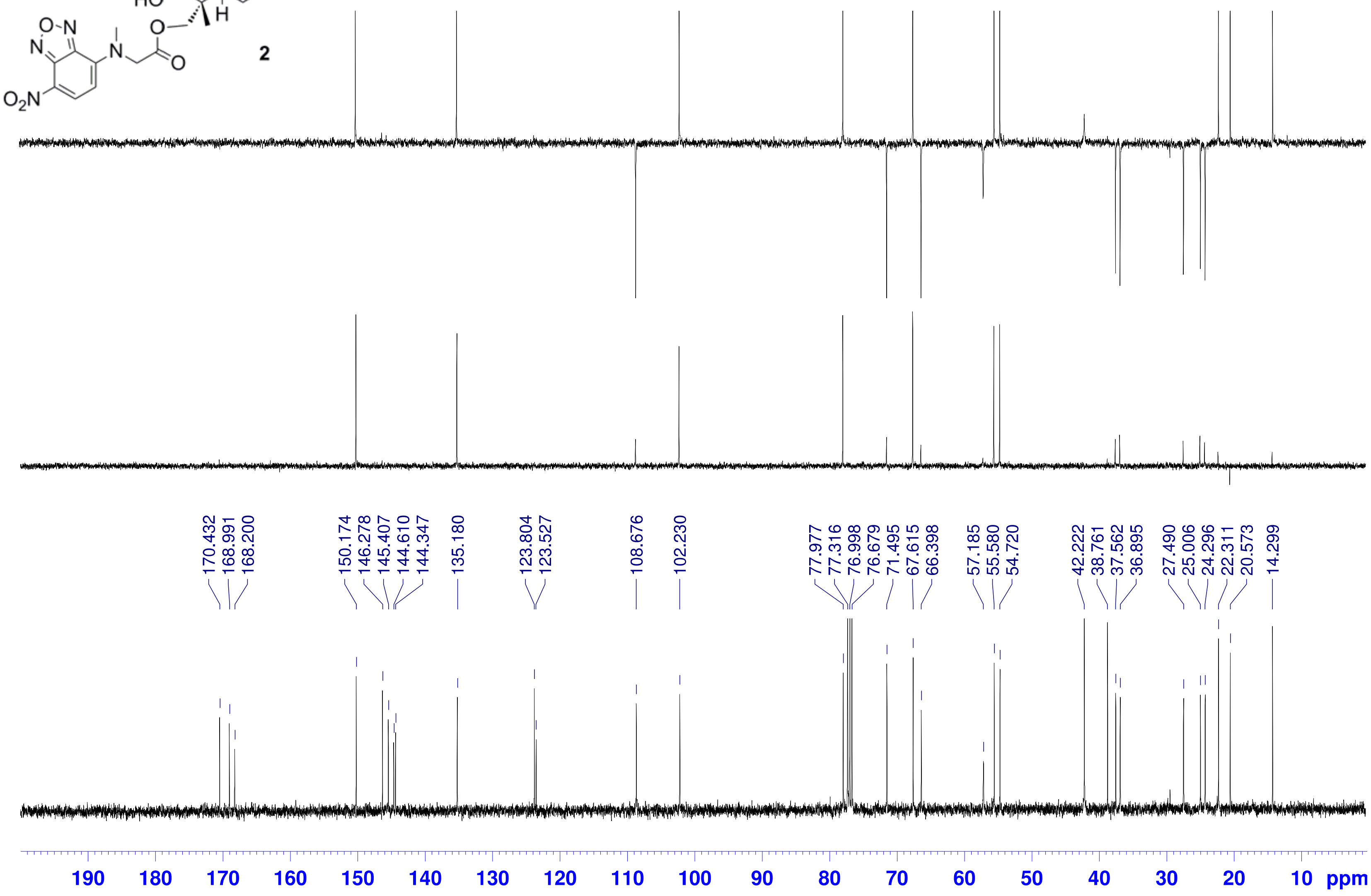

Supplement: S1 Fig — (PDF) [file pone.0152770.s001.pdf]
