# Supplementary material for: Development of a Bifunctional Andrographolide-Based Chemical Probe for Pharmacological Study
Source: PLoS One. 2016 Apr 1;11(4):e0152770. doi: 10.1371/journal.pone.0152770 (PMC4818061; doi:10.1371/journal.pone.0152770)
Supplement: S2 Fig — (PDF) [file pone.0152770.s002.pdf]

S2 Fig. The uptake kinetics of ANDRO-NBD in MDA-MB-231

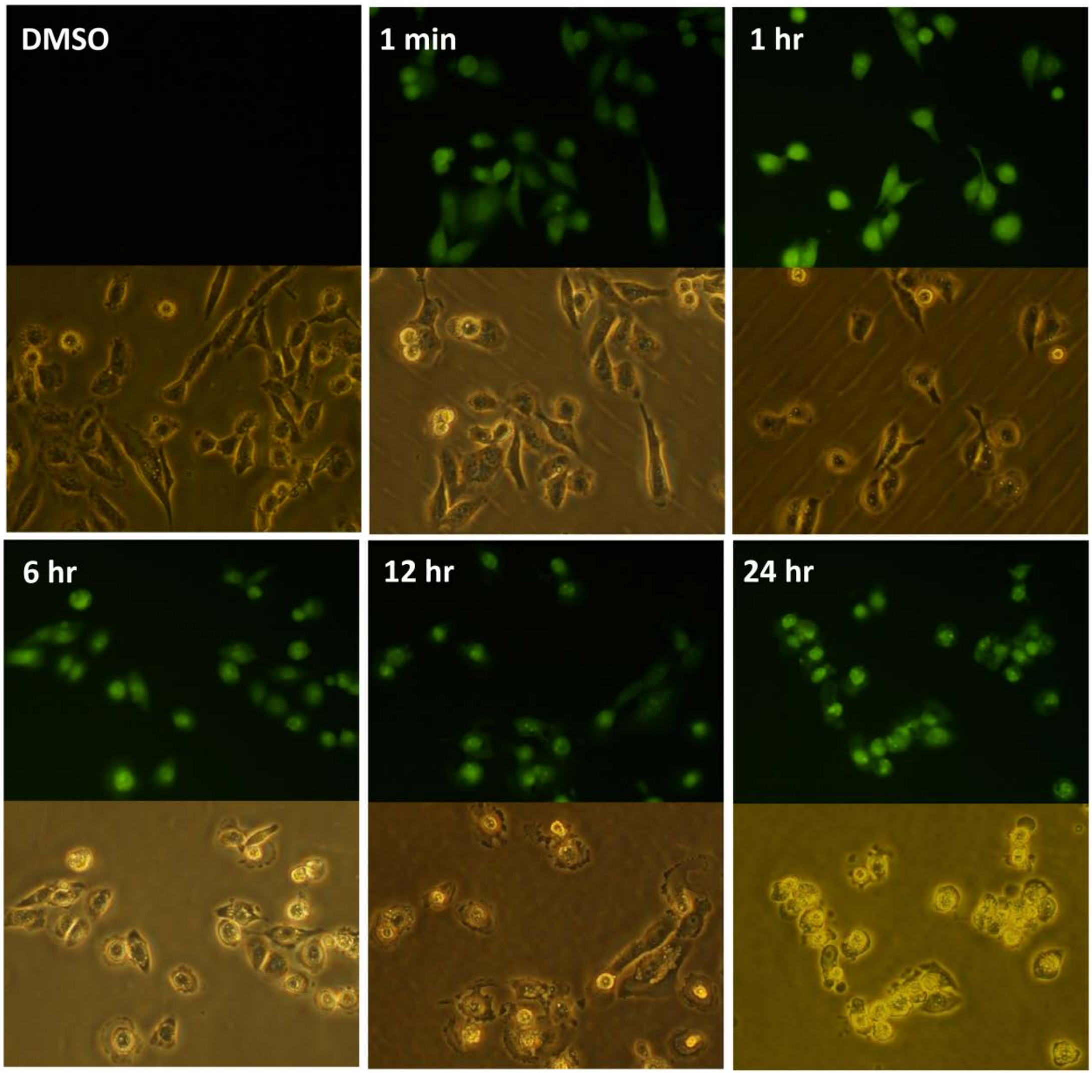

MDA-MB-231 cells (5x 10^4) in MP-24 plates were treated with vehicle (0.1% DMSO) or ANDRO-NBD 10 μM for indicated time periods. The images were photographed using a phase-contrast microscope with or without fluorescence illuminator.
