# Supplementary material for: Development of a Bifunctional Andrographolide-Based Chemical Probe for Pharmacological Study
Source: PLoS One. 2016 Apr 1;11(4):e0152770. doi: 10.1371/journal.pone.0152770 (PMC4818061; doi:10.1371/journal.pone.0152770)
Supplement: S3 Fig — (PDF) [file pone.0152770.s003.pdf]

S3 Fig. Pretreatment of andrographolide significantly reduced ANDRO-NBD-mediated fluorescence

(A)

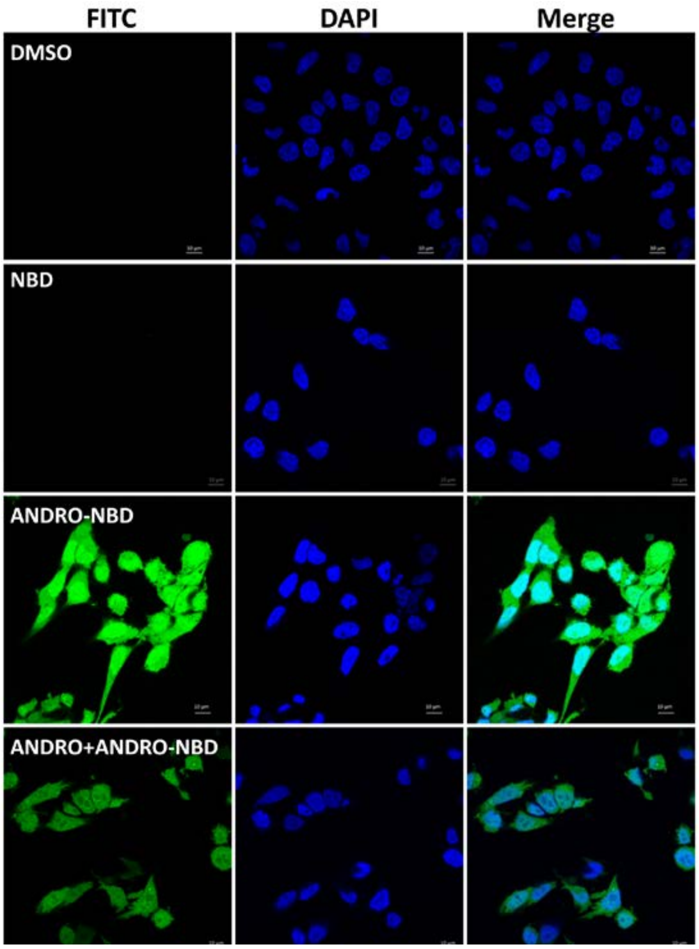

(B)

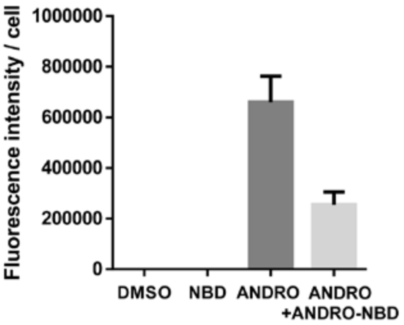

The ts-v-Src cells (1 x10^5) were seeded in MP-12 plates and grown at 35°C overnight. The cells were then treated with ANDRO (30 µM) or 0.1% DMSO for 6 hours, followed by ANDRO-NBD treatments (1 µM) for 10 min. Then medium was removed, and cells were washed with PBS and fixed by 4% formaldehyde. (A) The cellular image was photographed with a fluorescent confocal microscope (ZEISS LSM880, Germany) under same exposure time. (B) Images from three independent fields were quantified using MetaMorph (Molecular Devices, USA).
